# PARP (Poly ADP‐ribose Polymerase) Family in Health and Disease

**DOI:** 10.1002/mco2.70314

**Published:** 2025-09-01

**Authors:** Pengyuan Lei, Wenfeng Li, Jinhua Luo, Nanxin Xu, Yahe Wang, Dafei Xie, Hua Guan, Bo Huang, Xin Huang, Pingkun Zhou

**Affiliations:** ^1^ Beijing Key Laboratory for Radiobiology Department of Radiation Biology Beijing Institute of Radiation Medicine Beijing China; ^2^ College of Public Health University of South China Hengyang Hunan China; ^3^ Department of Occupational and Environmental Health Xiangya School of Public Health Central South University Changsha Hunan China; ^4^ Hengyang Medical School University of South China Hengyang Hunan China

**Keywords:** PARP family, physiological functions, pathological mechanisms, DNA damage repair, metabolic regulation, PARP‐targeted therapy

## Abstract

The poly(ADP‐ribose) polymerase (PARP) family consists of 17 members of nicotinamide adenine dinucleotide (NAD⁺)‐dependent enzymes that regulate key biological processes by catalyzing adenosine diphosphate (ADP)‐ribosylation, either poly(ADP‐ribosyl)ation (PARylation) or mono(ADP‐ribosyl)ation (MARylation). These biological processes encompass DNA repair, metabolism, telomere maintenance, and immune responses. Based on structural and functional features, the PARP family is classified into subcategories, such as DNA‐dependent PARPs, Tankyrase, CCCH‐type PARPs, MacroPARPs, and atypical PARPs. These enzymes dynamically maintain genome stability through mechanisms, including base excision repair and homologous recombination, while also regulating telomere dynamics and metabolic pathways. Dysregulation of PARP activity is implicated in the pathogenesis of diverse human diseases. Though PARP inhibitors have gained therapeutic interest in oncology, their wider roles in nononcological conditions, such as neurodegenerative diseases, cardiovascular disorders, and viral infections, remain poorly defined. This review elucidates the unique structural features of PARP family members and describes their multiple roles under physiological and pathological conditions, thus providing insights into treatment strategies. Additionally, it summarizes the advances and challenges in PARP‐targeted therapies and explores future directions for innovative therapeutic approaches. The findings may serve as a valuable resource for informing both clinical research and drug development.

## Graphical abstract

PARP family orchestrates ADP‐ribosylation to regulate genomic stability (DNA repair, telomeres) and metabolism (NAD⁺, glucose/lipid). Physiological roles sustain health; dysregulation drives pathologies: cancer (DNA repair addiction), neurodegeneration (parthanatos), cardiovascular disorders (hypertrophy/fibrosis), inflammation (dual role), and viral infections (antiviral defense vs. viral hijacking). PARP inhibitors show clinical promise, yet tissue‐specificity and resistance demand innovative strategies. Image created with BioRender.com, with permission.

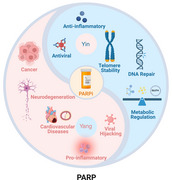

## 1 Introduction

The poly(ADP‐ribose) polymerase (PARP) family is a group of nicotinamide adenine dinucleotide (NAD⁺)‐dependent enzymes that catalyze ADP‐ribosylation, thus regulating key biological processes, such as DNA repair, genome stability, metabolism, and immune responses [1]. Since the discovery of PARP1 in 1963, researchers have extensively identified 17 members in humans (PARP1–16). Among them, PARP5A and PARP5B—also termed as Tankyrase‐1 (TNKS1) and Tankyrase‐2 (TNKS2), respectively—are marked by their distinctive ankyrin repeat domains (ARDs) flanking the catalytic sites. They exhibit unique structural and functional properties from other members [2, 3, 4, 5, 6, 7, 8, 9, 10, 11]. Based on structural and functional roles, the PARP family is broadly classified into five subgroups, namely DNA‐dependent PARPs, Tankyrases, CCCH‐type PARPs, MacroPARPs, and atypical members [2, 3, 4, 5, 12]. Of them, PARP1 is the most widely studied because it orchestrates DNA damage repair through base excision repair (BER) and homologous recombination (HR) [13, 14, 15, 16, 17, 18, 19, 20, 21]. In contrast, Tankyrases (PARP5A/5B) regulate telomere dynamics and Wnt signaling [22, 23, 24, 25]. Beyond their canonical roles in genome stability, emerging studies have highlighted PARPs as key modulators of metabolic reprogramming and inflammatory cascades [26, 27, 28, 29, 30, 31, 32, 33, 34, 35, 36], underscoring their multifaceted contributions to cellular processes.

*PARPs play a dual role*: they maintain genomic integrity and telomere stability under homeostasis through rapid DNA lesion repair [13, 14, 22, 23, 24, 25]. Nevertheless, they can exert detrimental effects in pathogenic conditions. PARP overactivation in pathological states causes NAD⁺ depletion, exacerbates inflammation, and promotes resistance to cancer therapies [26, 35, 36, 37, 38]. This paradox is exemplified by PARP1. PARP inhibitors (PARPi) induce synthetic lethality in BRCA‐deficient cancers [19, 20]. In contrast, excessive PARP1 activation drives neuronal death in neurodegenerative diseases [39, 40] and metabolic dysfunction in diabetes [41]. PARPi, such as olaparib, has achieved clinical success in BRCA1/2‐deficient cancers [19, 20]. However, the mechanisms underlying PARP involvement in nonmalignant pathologies, such as cardiovascular disorders and viral infections, remain underexplored [41, 42, 43, 44, 45, 46, 47].

This review synthesizes existing knowledge on the PARP family, emphasizing its structural diversity, catalytic mechanisms, and context‐specific biological roles. It systematically analyzes how various PARP enzymes regulate genome stability, metabolic homeostasis, and immune responses, while also dissecting their pathogenic contributions to cancer, neurodegenerative diseases, and inflammatory disorders. Furthermore, it critically evaluates the clinical potential and limitations of PARP‐targeted therapies, including catalytic inhibitors, allosteric modulators, and nanodelivery systems. By addressing knowledge gaps, such as tissue‐specific PARP interactomes and resistance mechanisms, this review aims to promote the development of precision‐based strategies for targeting PARPs in disease management.

## 2 Overview of the PARP Family and its Biological Functions

This section provides a comprehensive overview of the PARP family, focusing on its classification, structural features, catalytic mechanisms, and biological functions. The 17 members of the PARP family utilize NAD**^+^** as a substrate to catalyze either mono‐ or poly(ADP‐ribosyl)ation (MARylation/PARylation) of target proteins.

### 2.1 Classification and Structural Characteristics

**FIGURE 1:**
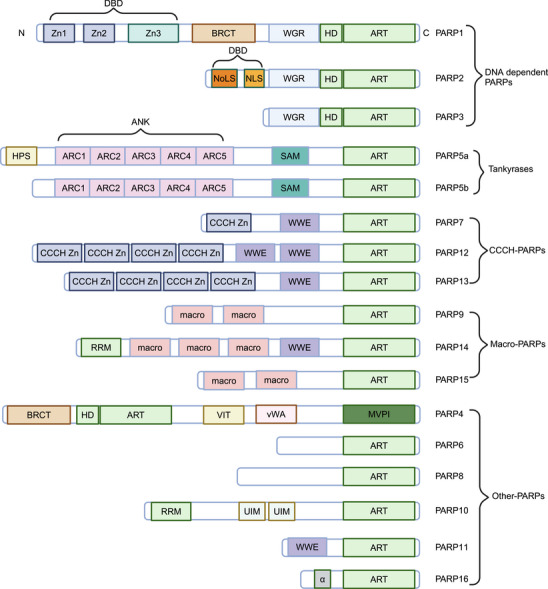
PARP family members and their structural domains. The domain architecture of PARP family members, including DNA‐dependent PARPs, Tankyrases, CCCH‐PARPs, macro‐PARPs, and other‐PARPs. *Abbreviations*: PARP, poly (ADP‐ribose) polymerase; CCCH, cysteine‐cysteine‐cysteine‐histidine motif. Image created with BioRender.com, with permission.

The PARP family transfers ADP‐ribose units from NAD⁺ to themselves or target proteins, either as branched chains (poly‐ADP‐ribosylation) or single units (mono‐ADP‐ribosylation), thereby regulating cellular responses. All PARPs contain a conserved C‐terminal catalytic domain (CAT), which regulates NAD⁺‐dependent poly(ADP‐ribose) (PAR) chain synthesis. Contrarily, their N‐terminal domains (NTDs) vary considerably, determining substrate specificity and subcellular localization. To date, 17 family members have been identified in humans, including PARP1 to 16 and Tankyrase‐1/2 (Figure 1). Importantly, all members do not show ADP‐ribosyltransferase activity [1]. Based on their domain composition and functional roles, PARPs are primarily classified into the following subcategories.

#### 2.1.1 DNA‐Dependent PARPs

This subcategory includes PARP1, PARP2, and PARP3, which contain DNA‐binding zinc finger domains and directly respond to DNA damage. Their CATs share 60% structural similarity, but their NTDs differ substantially. Particularly, PARP2 and PARP3 lack the zinc finger motifs and the BRCA1 C Terminus (BRCT) domain [48].

*PARP1 consists of three functional domains*: the N‐terminal DNA‐binding domain (containing two zinc finger motifs), the central auto‐modification domain (BRCT domain), and the C‐terminal CAT. The Zn1 domain governs its catalytic activity, the Zn2 domain specifically recognizes DNA damage sites [2], whereas the Zn3 domain, exclusive in PARP1, orchestrates its conformational rearrangement during activation [49]. The central auto‐modification domain contains a BRCT motif and flanking glutamic acid [50] or lysine residues [51], which serve as auto‐ADP‐ribosylation sites. Adjacent to the BRCT domain is the tryptophan–glycine–arginine (WGR) domain [52] and the ADP‐ribosyltransferase CAT (ART CAT) [53]. The CAT is a characteristic structure and is involved in activities related to the formation, extension, and branching of ADP‐ribose conjugates [54].

PARP2 shares 69% homology with PARP1 [55, 56] and can be divided into several functional regions: a DNA binding domain (DBD), a WGR domain, and a CAT. The DBD contains a nuclear localization sequence and a nucleolar localization sequence. Contrarily, PARP3 contains only the WGR domain and CAT in its C‐terminal region [57].

#### 2.1.2 Tankyrases

This subcategory includes PARP5A (Tankyrase1) and PARP5B (Tankyrase2), which contain ARDs and are involved in telomere length maintenance [58] and regulation of the Wnt signaling pathway [59]. Tankyrase is primarily composed of four parts: a C‐terminal PARP CAT, a sterile alpha motif (SAM) domain, an ARD central region (a long amino acid sequence composed of five tandem subunits), and an N‐terminal HPS domain (containing His, Pro, and Ser residues) [3]. The fundamental structural difference between the two isoforms is the absence of the HPS domain in Tankyrase2.

#### 2.1.3 CCCH‐Type PARPs

This subcategory comprises PARP7, PARP12, and PARP13. These enzymes contain three cysteines and one histidine zinc finger motif, as well as a tryptophan–tryptophan–glutamate (WWE) domain. PARP7 contains a single CCCH‐type zinc finger motif. PARP12 is structurally similar to PARP7 but contains multiple CCCH‐type zinc finger motifs. PARP13 exists as two isoforms, namely PARP13.1 and PARP13.2. Neither isoform shows catalytic ART activity. PARP13.1 contains a CAT similar to that of PARP1, but it lacks essential residues for ART activity. In contrast, PARP13.2 completely lacks the CAT [4]. Both isoforms include four zinc finger motifs involved in RNA binding.

#### 2.1.4 MacroPARPs

This subcategory includes PARP9, PARP14, and PARP15; it is characterized by a highly conserved macrodomain, approximately 190 amino acids long, which shows strong affinity for ADP‐ribose [60, 61].

PARP9 contains two macrodomains but lacks ART activity because of the absence of key residues. PARP14 contains three macrodomains, a WWE domain, and an RNA recognition motif, which allows mono‐ADP‐ribosylation of targets [62, 63]. PARP15 contains two macrodomains.

#### 2.1.5 Atypical PARPs

Because of structural complexity, these PARPs cannot be classified into the four subcategories. PARP4 shares one‐third similarity in the NTD with other family members. Its similarity with PARP1 includes a BRCT domain, a WGR domain, and a CAT [12]. PARP6 contains a highly conserved cysteine‐rich domain, which is central to neurodevelopment [7]. PARP10 contains a PCNA‐interacting protein motif (PIP‐box), which is crucial for the localization of PARP10 to single‐strand DNA breaks (SSBs) and its function in DNA repair [8]. PARP11 contains a spherical WWE domain, similar to PARP12, and helps in combating viral infections [9, 10]. PARP16 is a tail‐anchored membrane protein in the endoplasmic reticulum (ER); it contains a hydrophobic transmembrane domain and is primarily involved in regulating ER stress, angiogenesis, cellular aging, and apoptosis [64, 65].

The 17‐member PARP family centers on a conserved C‐terminal CAT, regulating substrate specificity and subcellular localization via diverse NTDs. Classified into DNA‐dependent PARPs, Tankyrase, CCCH‐type PARPs, MacroPARPs, and atypical PARPs, PARPs contribute to DNA repair, telomere length maintenance, viral RNA degradation, ADP‐ribose recognition, neurodevelopment, and ER stress regulation. Some members (e.g., PARP13) lack enzymatic activity but remain functionally critical through non‐CATs. This structural and functional specificity underpins the core role of PARPs in cellular homeostasis and pathogenesis.

### 2.2 Catalytic Activity and Mechanism of the PARP Family

The core enzymatic activity of PARP is to catalyze NAD^+^ hydrolysis, yielding ADP‐ribosyl groups that are transferred to glutamate, aspartate, or lysine residues on target proteins, such as histones, DNA repair enzymes, or PARP themselves. The addition of a single ADP‐ribose group is known as MARylation, but the addition of multiple ADP‐ribose groups to form linear or branched PAR chains is known as PARylation.

#### 2.2.1 DNA‐Dependent PARPs

During DNA damage, PARP1 binds to the damage site through its DNA‐binding domain, triggering a series of structural changes that activate its catalytic function. Activated PARP1 uses NAD^+^ as an ADP donor to conduct PARylation modifications on target proteins, thus mediating the recruitment of DNA repair factors and chromatin remodeling, finally promoting DNA repair [52].

PARP1 and PARP2 have complementary functions. PARP1 is primarily involved in repairing SSBs, whereas PARP2 additionally contributes to DNA repair and is central to metabolism and inflammatory response [66]. Upon DNA damage, PARP1 is recruited rapidly and instantaneously at the repair site, whereas PARP2 is recruited slowly and persistently [67]. PARP1 primarily binds to SSBs, whereas PARP2 shows a higher affinity for gaps formed by nucleotide loss [68].Additionally, the accumulation of PARP2 at the damage site and the recruitment of X‐ray repair cross‐complementing protein 1 (XRCC1) depend on PARP1 activity [69].

PARP3 primarily functions as a mono‐ADP‐ribosyltransferase. Moreover, it facilitates the repair of DNA double‐strand break (DSBs) repair together with PARP1. Mice lacking both PARP3 and PARP1 exhibit significantly increased sensitivity to ionizing radiation [70]. Additionally, PARP3 forms protein complexes with nuclear mitotic apparatus protein [71] and PARP5A [72], contributing to mitotic spindle formation and genome stability.

#### 2.2.2 Tankyrases

Tankyrases, including PARP5A and PARP5B, contain ARD and SAM domains. Apart from regulating DNA repair, they are central to telomere length maintenance and Wnt signaling. Tankyrase levels are regulated by the PAR‐binding E3 ubiquitin ligase RNF146, which regulates the K48‐linked polyubiquitination and proteasomal degradation of Tankyrase and related partners [73].

Telomeric repeat‐binding factor 1 (TRF1) is a negative regulator of telomerase function and can bind to telomeric DNA, reinforcing telomere structure [74]. Tankyrase binds to TRF1 through ADP‐ribosylation, which is further degraded by FBXO4 or RLIM/RNF12, leading to telomere complex disassembly and promoting telomere extension [75].This process is regulated by polo‐like kinase 1 (Plk1)‐mediated Tankyrase phosphorylation, which enhances its interaction with TRF1 and increases telomere stability. In contrast, Plk1 mediates the mutation of Tankyrase phosphorylation sites, thus decreasing its distribution at telomeres and spindle poles [76]. Fanconi anemia group D2 protein can inhibit TRF1 ADP‐ribosylation, and its absence promotes telomere extension [77]. In addition, Tankyrase interacts with Axin to regulate the Wnt pathway [78].

#### 2.2.3 CCCH‐Type PARPs

PARP7 is a mono‐ADP‐ribosyltransferase that can modify itself, core histones, and transcription factors. PARP7 is primarily located in the nucleus. Upon viral infection, PARP7 translocates to the cytoplasm, where its CCCH zinc finger domain binds viral RNA, inducing its degradation [79]. During tumor suppression, PARP7 negatively controls oncogenic transcription factors. It forms nuclear bodies in an ADP‐ribosylation‐dependent manner, which activate E3 ubiquitin ligases, namely HUWE1 and hypoxia‐inducible factor 1‐alpha (HIF‐1α). By accelerating HIF‐1α ubiquitination and degradation, it inhibits tumor growth and the Warburg effect in breast and colon cancer xenografts. Contrarily, in cancer cells, PARP1 promotes glycolysis by activating HIF‐1α under hypoxia [80].

PARP12 exerts numerous antiviral effects by inhibiting the translation of viral and cellular proteins. Moreover, PARP12 localizes to cellular stress granules [81]. During oxidative stress and viral infection, PARP1 gets activated, synthesizes PAR chains, and releases them into the cytoplasm. Subsequently, PARP12 binds to these chains, detaches from the Golgi membrane, and enters the cytoplasm to combine with stress granules, thus generating protective responses.

Both PARP13 isoforms, that is, PARP13.1 and PARP13.2, can bind to viral RNAs and target them for degradation through the cellular mRNA decay mechanism. However, PARP13 can bind to cellular RNAs and regulate transcription in healthy cells [82, 83].

#### 2.2.4 MacroPARPs

PARP9 serves as a noncanonical sensor of viral RNA, binding directly and recruiting the p85 subunit of phosphoinositide 3‐kinase (PI3K) and protein kinase B gamma (AKT3). This process promotes the phosphorylation of interferon regulatory factor 3 (IRF3) and IRF7, thus driving the transcription of type I interferon (IFN‐I) [84]. Although PARP9 lacks ART activity, it can form a complex with deltex E3 ubiquitin ligase 3‐like (DTX3L) (a histone E3 ligase involved in DNA repair) and PARP14 to regulate interferon‐induced ADP‐ribosylation [85].

PARP14 shows mono‐ADP‐ribosyltransferase activity and enhances interleukin‐4 (IL‐4)‐induced gene expression by interacting with signal transducer and activator of transcription 6 in B and T cells [62]. PARP15, in conjunction with PARP5, PARP12, PARP13, and PARP14, coordinates stress granule assembly as a dynamic response to cellular stress [86].

Taken together, PARPs hydrolyze NAD⁺ to generate ADP‐ribosyl groups, transferring them to target proteins via MARylation or PARylation. Different subclasses exhibit functional specialization:

- DNA‐dependent PARPs primarily drive DNA damage repair.
- Tankyrase regulates telomere elongation and Wnt signaling.
- CCCH‐type PARPs are involved in antiviral degradation and stress responses. MacroPARPs participate in interferon activation and immune signaling.

Collectively, these enzymes maintain cellular homeostasis in DNA repair, telomere stability, antiviral defense, and signaling.

## 3 Role of the PARP Family in Human Health

This section elucidates the dual role of PARPs in preserving genome stability and controlling cellular metabolism. PARP1/PARP2 drive DNA repair by orchestrating SSBs repair via PAR‐dependent recruitment of repair factors, such as XRCC1. Moreover, they control DSBs repair by balancing high‐fidelity HR and error‐prone nonhomologous end joining (NHEJ). Tankyrases maintain telomere integrity. In metabolism, PARP1 acts as a major NAD⁺ consumer; PARP1 overactivation depletes NAD⁺, impairs mitochondrial function, disrupts glycolysis, and affects lipid metabolism. Furthermore, Tankyrases regulate insulin‐dependent glucose uptake. Overall, PARP dysfunction contributes to cancer, metabolic disorders, and aging.

### 3.1 PARP Family Maintains Genomic Stability

#### 3.1.1 Core Drivers of SSBs Repair

Genome stability relies on an efficient DNA repair network. SSBs are the most common types of DNA damage, typically repaired through the BER pathway. PARP1 and PARP2 are central to this process.

PARP1 recognizes SSBs, disrupted phosphodiester bonds, such as via its N‐terminal three zinc finger (ZF1–3) domains, triggering conformational changes that activate the CAT. Activated PARP1 uses NAD^+^ to catalyze ADP‐ribosylation and synthesize long‐chain PAR polymers at the damage site [13]. This modification relaxes chromatin structure, increasing access to repair proteins, such as XRCC1 and DNA ligase III (LIG3) through charge attraction and domain interactions [52, 87]. XRCC1 is a scaffold protein in the BER pathway, and its function is highly dependent on PARP1's PARylation. Its BRCT domain directly binds to PAR chains, enabling rapid enrichment at the damage site. XRCC1 bridges additional repair proteins, including polynucleotide kinase 3'‐phosphatase, DNA polymerase β (Polβ), and LIG3 through its NTD, middle domain, and C‐terminal domain (CTD), forming a “repairosome” that ensures sequential gap processing, filling, and ligation [15, 16, 88]. PARP1's PARylation promotes XRCC1 recruitment, which stabilizes the PARP1–DNA complex, extending its activity and forming a synergistic amplification effect [17, 89]. LIG3 is the only DNA ligase that regulates the BER pathway, and its function depends on its interaction with XRCC1. LIG3's zinc finger domain binds to the CTD of XRCC1, preventing proteasomal LIG3 degradation. PAR chains enhance LIG3's affinity for DNA, improving ligation efficiency. Additionally, Sirtuin 6 (SIRT6) contributes to BER. Unlike its regulatory roles in other contexts, SIRT6 does not modulate the activation of PARP1 or PARP2. Instead, it influences DNA repair by regulating the recruitment of XRCC1, thereby reducing Polβ abundance at DNA damage sites [16].

**FIGURE 2:**
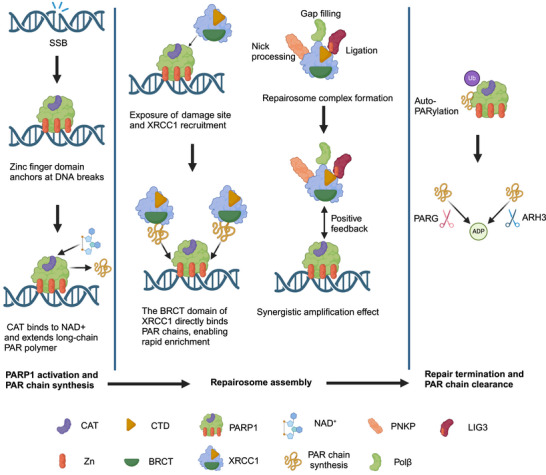
Role of PARP1 in single‐strand break repair. PARP1 recognizes SSBs via ZF1‐3, activates CAT to synthesize PAR chains, relaxes chromatin, and recruits XRCC1 and the repair complex (PNKP/Polβ/LIG3). The interaction between XRCC1 and PARP1 amplifies the repair signal. PARP1 undergoes self‐PARylation, triggering ubiquitination and degradation. PAR chains are removed by PARG (rapidly) and ARH3 (slowly), ensuring genomic stability. *Abbreviations*: PARP, poly (ADP‐ribose) polymerase; XRCC1, X‐ray repair cross‐complementing protein 1; SSBs, single‐strand breaks; CAT, catalytic domain; PNKP, polynucleotide kinase phosphatase; LIG3, DNA ligase III; Polβ, DNA polymerase beta; PARG, poly(ADP‐ribose) glycohydrolase; and ARH3, ADP‐ribosylhydrolase 3. Image created with BioRender.com, with permission.

To prevent excessive NAD^+^ consumption and uncontrolled repair signaling, PARP1 utilizes negative feedback through its PARylation [17]. The negatively charged PAR chain repels PARP1 from the DNA, promoting its dissociation from the damage site. Self‐PARylation recruits E3 ubiquitin ligases (such as checkpoint with forkhead and ring finger domains), mediating the ubiquitination and proteasomal degradation of PARP1, terminating the repair signal. Poly(ADP‐ribose) glycohydrolase (PARG) rapidly degrades PAR chains, which resets the chromatin to its original state and prevents continuous repair activation [90, 91, 92, 93]. Additionally, ADP‐ribosylhydrolase 3 degrades PAR chains but shows a different structure and slower catalytic activity than PARG [94]. The removal of PAR chains marks the completion of SSBs repair, facilitating the cell to return to homeostasis. The catabolic activity of PARG forms a dynamic equilibrium with the synthetic activity of PARP1, ensuring precise and timely DNA repair (Figure 2).

The FAcilitates Chromatin Transactions complex is composed of structure‐specific recognition protein 1 (SSRP1) and suppressor of Ty 16. It is central to maintaining genome integrity and chromatin remodeling. SSRP1 serves as a histone H2A/H2B chaperone during DNA transcription, replication, and repair. SSRP1 is recruited to SSBs in a PARP‐dependent manner and retained at the damage site through interaction with XRCC1's N‐terminus, increasing access to chromatin and facilitating SSBs repair [95]. PARP2 partially compensates for the lack of PARP1. PARP2 binds to DNA through its WGR domain and shows greater sensitivity to specific types of SSBs, such as 5′‐deoxyribose phosphate sites. It modifies histones H1 and H2B rather than the broad substrate spectrum of PARP1; however, its catalytic efficiency remains low [68].

#### 3.1.2 Regulatory Hub of DSBs Repair

DSBs are the most lethal type of DNA damage, and their repair necessitates a balance between high‐fidelity HR and rapid, error‐prone NHEJ. The PARP family, particularly PARP1 and PARP2, plays a crucial role in DSBs repair through the dynamic PARylation of chromatin and related proteins. They serve as a core regulatory hub, coordinating the selection of repair pathways, the assembly of repair complexes, and the transmission of DNA damage signals [87].

HR occurs during the S and G2 cell cycle phases and uses the sister chromatid as a template for high‐fidelity repair. After recognizing DSBs, PARP1 rapidly catalyzes local PAR chain synthesis at the damage site, mediating the relaxation of chromatin structure and exposing the DNA ends. PAR chains act as scaffold to recruit ataxia–telangiectasia mutated kinase, MRE11 nuclease, and the BRCA1 complex, initiating DNA end resection and generating single‐stranded DNA (ssDNA) regions necessary for HR [18]. PARylation of BRCA1's BRCT domain enhances its interaction with BRCA1‐associated RING domain protein 1, promoting the assembly of the BRCA1–PALB2–BRCA2 complex, which guides RAD51 nucleation on ssDNA. This process initiates homologous pairing and strand invasion [96].

In replication‐related DSBs, PARP1 stabilizes stalled replication forks by preventing MRE11‐mediated excessive resection and retaining sufficient ssDNA for HR. Additionally, PARP1 interacts with the TIMELESS protein to activate the ataxia–telangiectasia and Rad3‐related (ATR)–CHK1 pathway and restart replication [97]. The zinc finger transcription factor 1 E4F1 is rapidly recruited to DSBs in a PARP‐dependent manner, promoting ATR/CHK1 signaling, DNA end resection, and subsequent HR [98]. When BRCA1/2 mutations compromise HR, PARPi blocks PARP1 activity and traps the PARP–DNA complex, forcing cells to rely on substitute error‐prone NHEJ or microhomology‐mediated end joining. This results in catastrophic genomic rearrangements and even cell death [19, 20]. This “synthetic lethality” forms the core mechanism by which PARPi, such as olaparib and niraparib, facilitates the treatment of ovarian cancer, breast cancer, and prostate cancer (PCa). Furthermore, PARP1 inhibits the recruitment of the 53BP1–RIF1 complex, which suppresses DNA end resection and HR [99, 100]. 53BP1–RIF1 inhibition can restore HR in BRCA‐mutated cells, leading to PARPi resistance.

NHEJ directly joins the broken ends; however, it lacks a template and is prone to base loss or insertion errors. PARP1 restricts premature NHEJ activation via temporal and spatial regulation. In the S/G2 phase, PARP1 PARylation inhibits the binding of Ku70/80 (the NHEJ core factor) to DNA ends, ensuring HR [101]. Meanwhile, PAR chains shield DNA ends, preventing the premature recruitment of DNA‐dependent protein kinase catalytic subunit (a key NHEJ kinase), which is activated by SIRT2‐regulated deacetylation [102, 103]. In case of incomplete HR (such as the absence of sister chromatids), PARP1 activity decreases, and PAR chains are rapidly degraded by PARG. It reverses the inhibition of Ku70/80, thus initiating NHEJ [104]. Overexpression of TRABID can promote NHEJ repair by prolonging 53BP1 retention at the DSBs site [105]. Shieldin, a downstream 53BP1–RIF1 effector, inhibits DNA end resection and sensitizes BRCA‐deficient cells to PARPi [106].

DSBs synapsis is mediated by the DSBs sensor protein PARP1 via co‐condensing with DNA. It forms condensates at DSBs regions, and these condensates exert force to bring the DNA ends closer, facilitating ligation and preventing separation. In this process, FET proteins stabilize these PARP1–DNA condensates by binding to PAR, promoting DSBs repair [107]. When PARP1 function is impaired or inhibited, SSBs breaks accumulate, which leads to replication fork stalling and secondary DSBs. This can eventually lead to genome instability.

#### 3.1.3 Telomere Maintenance and Chromosome Stability

Telomeres are repetitive DNA sequences (TTAGGG) at chromosomal ends and associated protein complexes (termed Shelterin). Telomere length and structural integrity are crucial for maintaining chromosome stability. Tankyrases (PARP5A/PARP5B) modify TRF1 through PARylation, promoting telomerase approach to the telomere ends and maintaining the length [22]. Nonetheless, the relationship between Tankyrase expression and telomerase activity is still inconclusive. In the gastric cancer cell line SGC‐7901, Tankyrase inhibitors and telomerase activity inhibitors show a synergistic effect [23]. Increased Tankyrase expression correlates with raised telomerase activity and gastric cancer progression; however, a functional connection between telomerase activity and Tankyrase expression remains elusive [108]. In addition to telomere length maintenance, Tankyrase preserves the telomere structure [25].

Telomere capping requires classical DNA repair proteins, such as DNA‐dependent protein kinase (DNAPK) [109]. In cells lacking DNAPK, uncapped telomeres may be considered DSBs, leading to telomere‐telomere fusion or telomere‐DSBs fusion. DNAPK's protein stability depends on Tankyrase‐mediated ADP‐ribosylation. Reducing Tankyrase activity through RNA interference or chemical inhibitors leads to proteasomal DNAPK degradation, increasing sensitivity to ionizing radiation‐induced cell death, chromosome aberrations, and telomere fusion [110]. During mitosis, Tankyrase is localized to the spindle poles, telomeres, and the Golgi apparatus [111], where it contributes to the separation of sister chromatids and accurate mitosis [112]. In meiotic cells, Tankyrase binds to telomeres during the bouquet stage, maintaining telomere integrity [113]. Additionally, the absence of Tankyrase activity increases telomere fragility, causing chromosome end fusion or breakage [114].

The role of PARP1 in regulating telomerase activity remains controversial. PARP1 removal in mice or its inhibition in HeLa cells does not affect telomerase activity [115, 116]. However, small interfering RNA‐mediated PARP1 signaling reduces telomerase activity [117]. During chromatin structure regulation, PARP1 binds to nucleosomes in a NAD^+^‐dependent manner, without altering core histones or promoting nucleosome disassembly. In contrast, PARP1 induces chromatin changes in response to heat shock or other signaling pathways, possibly because of its interactions with transcription‐dependent nucleosomes [118]. Furthermore, PARP1 indirectly alters chromatin structure by regulating histone modification and chromatin remodeling enzymes [119]. PARP1 interacts with histone polyADP‐ribosylation factor 1 (HPF1) to catalyze histone ADP‐ribosylation, leading to chromatin relaxation.

### 3.2 PARP Involvement in Cellular Metabolism

#### 3.2.1 NAD^+^ Metabolism

**FIGURE 3:**
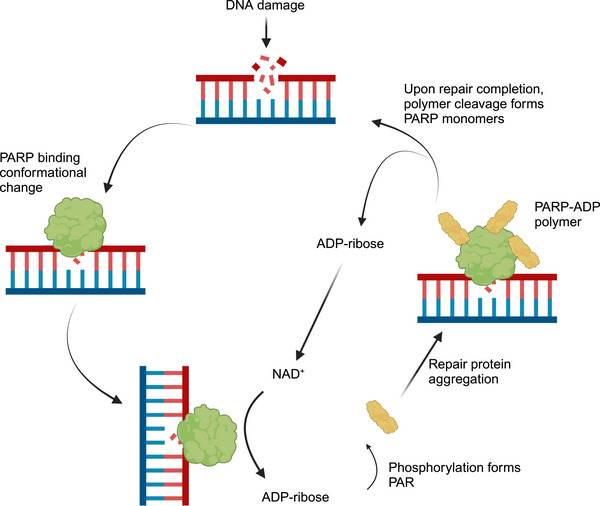
PARP1 and NAD^+^ metabolism regulation. PARP (particularly PARP1) is the primary cellular NAD⁺ consumer; it dynamically regulates NAD⁺ levels. *Abbreviations*: PARP, poly (ADP‐ribose) polymerase; NAD⁺, nicotinamide adenine dinucleotide. Image created with BioRender.com, with permission.

NAD^+^ is a central cofactor in redox reactions and energy metabolism. Moreover, it functions as an essential substrate for PARP catalytic activity. PARP1, specifically, is a major intracellular NAD^+^ consumer, dynamically regulating NAD^+^ levels and influencing metabolic pathways (Figure 3). PARP1 activation primarily leads to NAD^+^ decline, which results in cellular energy abnormalities [26]. The excessive activation of PARP1 in response to DNA damage reduces NAD^+^ levels and affects mitochondrial function and the selection of metabolic pathways. By modulating mitochondrial oxidative metabolism, PARP1 leads to paclitaxel‐induced neuropathic pain [27, 28].

Additionally, NAD⁺ depletion resulting from overactive PARP1 suppresses SIRT1‐dependent activation of the AMP‐activated protein kinase (AMPK) pathway, thereby impairing the cell's capacity to counteract metabolic stress [29, 120, 121]. Nonetheless, NAD^+^ can be generated via a salvage pathway. Increased PARP activity activates nicotinamide phosphoribosyltransferase (NAMPT), promoting the conversion of nicotinamide to NAD^+^, thus forming a metabolic compensation loop [122]. In chronic inflammatory diseases, such as psoriasis, NAMPT–PARP1 axis hyperactivation drives DNA damage in keratinocytes and facilitates parthanatos (a form of programmed cell death). Targeting the NAMPT–PARP1 axis or NAD⁺ replenishment reverses inflammatory phenotypes [123]. Similarly, in inflammatory pain models, NAMPT/PARP1 in the dorsal root ganglion mediates nociception via the nuclear factor kappa B (NF‐κB)/IL‐1 beta pathway. Moreover, NAMPT/PARP1 axis inhibition alleviates hyperalgesia [124].

#### 3.2.2 Sugar Metabolism

Hexokinase (HK), the enzyme that initiates glycolysis, catalyzes the conversion of glucose into glucose‐6‐phosphate, thereby starting the glycolytic pathway. PARP1 activation interferes with this process by consuming NAD^+^, thus affecting HK activity and disrupting glucose phosphorylation, which slows glycolysis. Glyceraldehyde‐3‐phosphate dehydrogenase (GAPDH), another key enzyme in glycolysis, is regulated by PARP1. PARP1 directly inhibits GAPDH activity via ADP‐ribosylation. Moreover, activated PARP1 in the cytoplasm leads to NAD^+^ degradation, further limiting GAPDH function and suppressing glycolytic flux [30]. The impact of PARP1 extends beyond the initial stages of glycolysis. The pyruvate dehydrogenase complex (PDC) converts pyruvate into acetyl‐CoA and channels it into the tricarboxylic acid (TCA) cycle. PARP1 regulates PDC subunits through polyADP‐ribosylation, potentially altering the fate of pyruvate and determining whether it enters the TCA cycle, is converted into lactate, or participates in gluconeogenesis [125]. Pyruvate supplementation alleviates cellular dysfunction caused by PARP1 activation, demonstrating pyruvate's role in glycolysis and energy metabolism [126]. Exogenous pyruvate maintains ATP production through PARP‐dependent glycolysis and PARP‐independent TCA cycle under high‐glucose conditions. PARPi, such as rucaparib, can restore ATP generation through glycolysis; however, it does not restore pyruvate dehydrogenase activity or mitochondrial ATP production [31].

Tankyrases (PARP5A/5B) contribute to glucose metabolism. Glucose transporter type 4 (GLUT4), a glucose transporter, is primarily found in glucose storage vesicles (GSVs). During elevated blood glucose levels, insulin secretion increases and induces the translocation of GSVs to the cell membrane. This process promotes the release of GLUT4 and stimulates glucose absorption from adipose and muscle tissues [127]. The insulin‐responsive aminopeptidase (IRAP), a cargo protein inside GSVs, mediates GLUT4 transport. IRAP knockdown can block GLUT4 translocation. Tankyrases bind to IRAP and regulate the intracellular distribution of both IRAP and GLUT4. Under insulin‐induced conditions, Tankyrase knockdown blocks GSV translocation and glucose uptake.

Kinesin family member 3A (KIF3A) is required for GSVs to respond to insulin‐induced translocation [128]. Tankyrase2 forms a complex with Axin and KIF3A to regulate insulin‐induced GLUT4 translocation. When insulin is lacking, the ADP‐ribosylation activity of Tankyrase2 causes the ubiquitination and degradation of this protein complex, thus inhibiting KIF3A function and GLUT4 translocation. Elevated insulin levels inhibit the ADP‐ribosylation activity of Tankyrase2, reducing glycosylation and ubiquitination and stabilizing the Tankyrase2 complex. This process promotes GLUT4 translocation [127].

PARP10 silencing induces glycolysis and mitochondrial oxidation, thereby causing a high metabolic state [129]. PARP14 supports glycolysis in lymphoma cells, but the underlying mechanism has not been elucidated [130]. It may promote glycolysis by interacting with HK and phosphofructokinase.

#### 3.2.3 Lipid Metabolism

PARP1 and PARP2 are central to the differentiation of adipocytes and lipid accumulation by modulating nuclear NAD^+^ levels. PARP1 activity influences the differentiation of adipocytes [32]. Specifically, nicotinamide mononucleotide adenylyltransferase 1 and 2 regulate the synthesis of nuclear NAD^+^ by competitively using nicotinamide mononucleotide, thereby affecting PARP1 activity. An increase in PARP1 activity inhibits adipocyte differentiation, whereas a decrease promotes its differentiation [33]. The sterol regulatory element‐binding protein 1c (SREBP1c)–PARP1 axis regulates adipocyte senescence. SREBP1c interacts with PARP1 and enhances its activity during DNA repair. In obesity, the absence of SREBP1c accelerates adipocyte senescence, recruiting immune cells to adipose tissues and triggering unhealthy tissue remodeling and insulin resistance [34]. In mouse models, obesity exacerbates postoperative cognitive dysfunction, with one key mechanism being PARP overactivation. For instance, PARP1 expression is significantly increased in the hippocampus of obese mice, accompanied by decreased NAD^+^/NADH ratio and SIRT1 expression. Hence, PARP1 activation may interfere with lipid and glucose metabolism and disrupt metabolic homeostasis in adipocytes by consuming NAD^+^ and inhibiting SIRT1 function [131].

## 4 Role of the PARP Family in Diseases and Clinical Applications

Extensive research has demonstrated the role of the PARP family in disease onset. The PARP family provides not only novel insights into disease mechanisms but also broad avenues for clinical diagnosis and treatment. From tumors and neurodegenerative diseases to cardiovascular diseases and inflammatory diseases, PARP members function as “molecular keys” that regulate the cellular microenvironment, thus facilitating precise diagnosis and targeted therapy. This section systematically elaborates on the key roles of the PARP family across different diseases and their translational value in clinical practice (Table 1).

**TABLE 1:** Disease‐specific roles and mechanisms underlying PARP family members.

Disease category	PARPs	Mechanism and role	References
Cancer	PARP1	In triple‐negative breast cancer, USP15 stabilizes PARP1 through deubiquitination → enhances base excision repair (BER); ER/PR/HER2 deficiency → ↑USP15–PARP1 interaction → promotes tumor growth and survival.	[39]
		In lung cancer, PARP1 activates the AMPK–mTOR pathway → enhances BDH1‐mediated autophagy → promotes cell proliferation/metastasis; BDH1 overexpression accelerates in vivo tumor growth.	[132]
		In colorectal cancer, PARP1 interacts with MARVELD1 to form a positive feedback loop → stabilizes PARP1 and ↑NAA50‐mediated acetylation → enhances genomic stability and chemoresistance.	[133]
		In B‐cell lymphoma, PARP1 deficiency → ↑proinflammatory response and Treg cell accumulation → accelerates c‐Myc‐driven lymphoma progression.	[134]
	PARP2	PARP2 deficiency remodels the bone microenvironment → Treg/Th1 imbalance → immune suppression → ↑bone metastasis.	[45, 47, 135]
	PARP4	Binds hnRNPM to suppress LUAD tumorigenicity; PARP4 deficiency causes splicing dysregulation → promotes tumor progression.	[136]
	PARP5	PARP5 deletion inhibits HNSCC tumor growth by activating ATR, depleting cancer stem cells, and impairing NHEJ repair.	[137]
	PARP6	HNSCC: SNHG1 inhibition ↑PARP6 → suppresses tumor growth/migration; HCC: Low PARP6 → HDM2‐mediated XRCC6 degradation → ↑Wnt signaling → promotes progression.	[138, 139]
	PARP7	Restricts the cytoplasmic accumulation of immunogenic nucleic acids in malignant/premalignant cells.	[140]
	PARP10	↑Replication fork stability → ↓replication stress → promotes tumor growth; Inhibits Aurora A kinase via mono‐ADP‐ribosylation → ↓EMT → suppresses metastasis.	[141, 142]
	PARP11	Hyperactivation in the immunosuppressive TME (e.g., adenosine) → β‐TrCP‐mediated IFNAR1 ubiquitination/degradation → ↓CD8^+^ CTL activity and survival.	[143, 144]
	PARP12	Stabilizes FHL2 and suppresses TGF‐β1 → inhibits HCC migration/invasion and metastasis.	[145]
	PARP16	ER stress activation → ARTC1 inactivates GRP78 via ADP‐ribosylation to trigger UPR; PARP16 ↑PERK/IRE1α activity → enhances stress response. PARP16 mediates CYB5R3‐dependent lung cancer cell death.	[146]
Neurodegenerative diseases	PARP1	Moderate PARP1 activation repairs DNA damage in early stages; with disease progression, pathological damage accumulation → PARP1 hyperactivation → triggers parthanatos and neuroinflammation → accelerates disease deterioration.	[39]
	PARP6	Loss of PARP6 catalytic activity impairs human neuronal function (mechanistic details require further study).	[147]
	PARP16	Acts as an RNA‐binding protein to stabilize APP mRNA → increases APP levels → promotes Alzheimer's disease pathology.	[65, 148, 149]
Cardiovascular diseases	PARP9	PARP9 overexpression antagonizes pirfenidone (the antifibrotic drug) → prevents Ang II‐induced downregulation of fibrosis markers (collagen I/III, α‐SMA).	[150]
	PARP10	The CHAPIR–PIWIL4 complex blocks METTL3‐mediated m6A methylation of PARP10 mRNA → ↑PARP10 → mono‐ADP‐ribosylation inhibits GSK3β → nuclear NFATC4 accumulation → promotes pathological hypertrophy.	[151]
	PARP15	ADP‐ribosylates JIP3 → inhibits p38 MAPK → maintains endothelial barrier integrity.	[136]
	PARP16	Activates the IRE1α–SXBP1–GATA4 pathway → promotes cardiac hypertrophy. Alleviates transverse aortic constriction/phenylephrine‐induced cardiac dysfunction and fibrosis.	[152]
Inflammatory diseases	PARP1	Crohn's disease: STC1 upregulation → binds to PARP1 → activates the JNK pathway → promotes oxidative stress‐related cell death and inflammation. Intervention: STC1 knockout or PARP1/JNK inhibition alleviates colitis. Tuberculosis: PZA inhibits PARP1 → reduces inflammation, but bactericidal efficacy is impaired in PARP1‐deficient mice. Adjunctive PARP1 inhibition may ↓lung lesions (or shorten treatment).	[35, 36]
	PARP2	Psoriasis: PARP2 silencing → ↑estradiol → inhibits NF‐κB → alleviates dermatitis. Colitis: PARP2 deficiency in T cells → (↓TNFα/IL‐17 + ↓oxidative stress/PARP1 activation) → suppresses intestinal inflammation.	[153, 154]
	PARP9	Proinflammatory factor → enhances IFNγ responses (macrophage‐mediated vascular diseases). Rheumatoid arthritis: Hypomethylation → ↑PARP9 mRNA → promotes Jurkat cell proliferation/activation.	[84, 155, 156]
	PARP12	Osteoarthritis: PARP12↑ → binds ISG15 → ↑MFN1/2 isoprenylation → inhibits PINK1/Parkin‐mediated mitophagy → promotes cartilage degradation.	[157]
	PARP14	Anti‐inflammatory factor → suppresses IFNγ responses and ↑IL‐4 responses. Antagonizes PARP9 in inflammatory vascular disease regulation.	[62]
Infectious & viral diseases	PARP1	DNA viruses induce RNS/DNA‐PK → PARP1 phosphorylation (Thr594) → cytoplasmic translocation → binds to and inhibits cGAS (Asp191) → suppresses antiviral immunity.	[158]
	PARP9	Recognizes dsRNA → activates the PI3K/AKT3 pathway → phosphorylates IRF3/7 → promotes type I interferon production. Also regulates PARP14 stability via the DTX3L complex.	[84, 159]
	PARP10	MARylation inhibits CHIKV nsP2 protease → blocks polyprotein processing → suppresses viral replication.	[160]
	PARP11	Cooperates with PARP12 → degrades ZIKV NS1/NS3 proteins (ISG‐dependent). Mono‐ADP‐ribosylates β‐TrCP → promotes IFNAR1 ubiquitination and degradation → inhibits IFN signaling (immune evasion mechanism).	[9, 161, 162]
	PARP12	Suppresses coronavirus replication. Binds to ISG15 → enhances MFN1/2 isoprenylation → inhibits mitophagy.	[163]
	PARP13	Restricts its own translation → regulates mRNA pool allocation → upregulates ISG acylation → maintains an antiviral cellular environment.	[164]
	PARP14	IFNγ‐induced → modulates anti‐inflammatory/pro‐IL4 responses. Its ADP‐ribosylation is hydrolyzed by SARS‐CoV‐2 Nsp3 → inhibits antiviral signaling (potential therapeutic target).	[159]

### 4.1 Role in Cancer and Targeted Therapy

Cancer development is a multistep process that involves acquiring sustained proliferative signaling, deregulated cellular metabolism, resistance to cell death, increased genomic instability, angiogenesis, tumor invasion, inflammation, replicative immortality, evasion of growth suppressors, and immune evasion [165]. PARP members contribute to tumor growth and regulate their microenvironment by participating in tumor progression.

**FIGURE 4:**
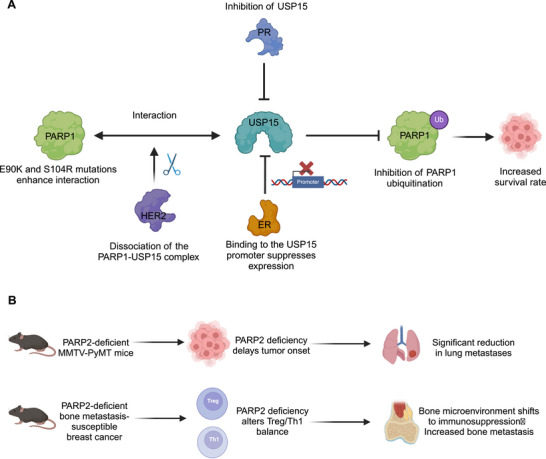
Role of PARP1 and PARP2 in breast cancer. (A) In breast cancer, two PARP1 mutations (E90K and S104R) enhance the interaction between PARP1 and USP15, inhibit PARP1 ubiquitination, and increase PARP1 levels, promoting tumor growth and survival. Specific loss of ER/PR/HER2 can increase PARP1 levels. (B) In PARP2‐deficient MMTV–PyMT mice, lung metastasis is significantly reduced. In bone metastasis models, PARP2 deficiency promotes an immunosuppressive microenvironment via Treg/Th1 imbalance, enhancing bone metastasis. *Abbreviations*: PR, progesterone receptor; PARP, poly (ADP‐ribose) polymerase; ER, estrogen receptor; HER2, human epidermal growth factor receptor 2; Th1, T helper type 1 cell; and Treg, regulatory T cell. Image created with BioRender.com, with permission.

As a core member, PARP1 plays a pro‐oncogenic role in various malignancies. However, PARP1 activity depends on the context, and PARP1 can exert both prosurvival and prodeath effects depending on the cellular environment and the nature and intensity of genotoxic stress. In triple‐negative breast cancer (TNBC), ubiquitin‐specific protease 15 (USP15) (a deubiquitinating enzyme) stabilizes PARP1, enhancing BER capacity and promoting tumor cell proliferation. Two PARP1 mutations (E90K and S104R) found in breast cancer enhance the interaction between PARP1 and USP15 and inhibit PARP1 ubiquitination, leading to abnormally elevated PARP1 levels. Furthermore, the estrogen receptor (ER), progesterone receptor (PR), and human epidermal growth factor receptor 2 (HER2) inhibit USP15‐mediated PARP1 stabilization through different mechanisms. ER binds to the USP15 promoter to suppress its expression, PR inhibits the deubiquitinating enzyme activity of USP15, and HER2 disrupts the PARP1–USP15 interaction (Figure 4A). The loss of all three receptors in TNBC increases PARP1 levels, resulting in enhanced BER capacity and increased TNBC survival rates [166].

In lung cancer, PARP1 mediates the AMPK–mechanistic target of rapamycin (mTOR) pathway, which regulates autophagy induced by β‐hydroxybutyrate dehydrogenase 1 (BDH1). Activation of the mTOR pathway significantly enhances BDH1‐mediated cell proliferation and metastasis. The intratumoral injection of BDH1 effectively promotes tumor growth in H460 xenograft mouse models [43]. In osteosarcoma (OS), marked by BRCA deficiencies and chromosomal instability, PARPi may reflect an effective therapeutic strategy. In two orthotopic OS mouse models, PARPi inhibited the growth of orthotopic tumors, regardless of BRCA status. However, PARPi treatment aggravated pulmonary metastasis in both models. Ezrin has been identified in OS metastasis; it serves as an interactive protein for PARP1. Ezrin phosphorylation was significantly promoted during PARP inhibition [41]. This finding underscores the duality of PARP1: though its hyperactivation supports tumor survival and DNA repair, its inhibition (via PARPi) can promote metastasis, potentially by disrupting cytoskeletal regulation and enhancing cell motility.

In colorectal cancer (CRC), MARVEL domain‐containing protein 1 (MARVELD1) mediates DNA damage response to maintain genome stability. PARP1 PARylates MARVELD1 at D102, D118, and D130. In turn, MARVELD1 stabilizes PARP1 by enhancing NAA50‐mediated acetylation, thus forming a positive feedback loop. MARVELD1 knockout mice and their embryo fibroblasts exhibit genome instability and a shorter PARP1 half‐life. Additionally, in patient‐derived xenograft models of CRC, MARVELD1 combines with PARP1 to promote resistance to genotoxic drugs and disrupts PARPi efficacy [44]. Further research revealed that NUDT13 suppresses PARP1‐mediated PKM1 PARylation, stabilizes PKM1 protein, and induces an oxidative phosphorylation phenotype in CRC cells, thereby significantly inhibiting tumorigenesis; Nudix motif‐targeted peptides can block CRC progression [167].

In hematologic malignancies, dysregulation of the c‐Myc oncogene, particularly its overexpression, is associated with aggressive tumor growth. PARP1 and PARP2 exert opposing effects in c‐Myc‐driven B‐cell lymphoma. They catalyze the synthesis and transfer of ADP‐ribose units to the amino acid residues of target proteins in response to DSBs, regulating the response to DNA damage. In an Em‐Myc mouse model, PARP1 deficiency accelerated lymphoma development. Moreover, it induced a proinflammatory response and increased regulatory T cells, which may facilitate immune evasion, leading to accelerated lymphoma development [45]. This contrasting effect of PARP1 deficiency (accelerating lymphoma) versus its prosurvival role in solid tumors highlights the tissue and context specificity of PARP1 function. Furthermore, during severe genotoxic stress, such as intensive radiotherapy or chemotherapy, excessive PARP1 activation leads to catastrophic depletion of cellular NAD^+^ and ATP, triggering parthanatos [168]. Hence, PARP1 hyperactivation reflects a prodeath signal in tumor growth. Thus, while chronic or dysregulated PARP1 activity promotes tumorigenesis and therapy resistance, acute PARP1 overactivation under therapeutic pressures can be harnessed to induce tumor cell death. This delicate balance underscores the importance of context when evaluating PARP1's role as either an oncogene or a tumor suppressor.

In the mouse mammary tumor virus–polyoma middle T antigen (MMTV–PyMT) mouse model of spontaneous breast cancer, PARP2 deficiency in the entire mouse or the breast delayed tumor onset, without affecting the growth rate. Selective PARP2 deficiency in the myeloid cell lineage did not affect tumor initiation. Compared with controls, PARP2‐deficient MMTV–PyMT mice and wild‐type (WT) mice implanted with PARP2‐deficient cells showed significantly reduced lung metastasis [46]. Conversely, in another syngeneic mouse model of bone metastasis‐prone breast cancer, PARP2 deficiency increased bone metastasis by altering the balance between regulatory T and T helper 1 cells, thereby converting the bone microenvironment into an immunosuppressive state [47] (Figure 4B).

In another syngeneic breast cancer model, the proficient AT‐3 breast cancer cell line was implanted into WT mice and those with T cell‐specific PARP2 deficiency. Compared with control mice, AT‐3‐induced tumor growth was significantly reduced in host mice, indicating the crucial role of PARP2 in regulating T cell responses in breast cancer. In a TNBC model, the selective degradation of PARP2 via proteolysis‐targeting chimeras (PROTACs) has therapeutic effects both in vitro and in vivo [169]. Gui et al. [170] reported that, in addition to its role in DNA damage repair, PARP2 is a key regulator of androgen receptor (AR) transcription. PARP2 interacts with the pioneer factor FOXA1 to promote AR recruitment to prostate‐specific enhancer regions across the genome. PARP2 expression is significantly higher in primary PCa tumors than in benign prostate tissues, with even higher expression in castration‐resistant prostate cancer (CRPC) tumors. Selectively targeting PARP2, either genetically or pharmacologically, to block its interaction with FOXA1 can weaken AR‐mediated gene expression and inhibit the growth of AR‐positive PCa cells. Next‐generation antiandrogen therapies work by inhibiting androgen biosynthesis (abiraterone) or blocking AR ligand binding (enzalutamide). Therefore, selectively inhibiting PARP2 may provide a novel therapeutic approach to inhibit AR activity by disrupting FOXA1 function [170]. PARP2 deficiency may affect specific c‐Myc‐driven blood malignancies [45]. The absence of PARP2 exacerbates replicative stress in preleukemic Eμ‐Myc B cells, increasing DNA damage, promoting cell death, and finally limiting the c‐Myc‐driven expansion of B cells [45].

Researchers have identified a significant, vault‐independent, tumor‐suppressive role of PARP4. Heterogeneous nuclear ribonucleoprotein M (hnRNPM) serves as a novel interaction partner for PARP4 and regulates the tumorigenesis of lung adenocarcinoma (LUAD). The commonality in splicing alterations caused by the loss of PARP4 and hnRNPM indicates that protein disruptions cause RNA splicing dysregulation, thus leading to LUAD pathogenesis. This study contributes to precision medicine by identifying clinically relevant modulators of tumorigenesis and necessitates targeting genetic mutations identified from tumor sequencing studies with their functional relevance. Beyond the existing driver mutations, alternative regulatory players should be explored, which may broaden the repertoire of LUAD therapeutic targets and biomarkers [136].

Despite changes in gene copy number of PARP5B in many cancers, the phenotypic impact of these alterations is largely unknown. To this end, PARP5B inactivation was described in carcinogen‐induced in vivo models of head and neck squamous cell carcinoma (SCC). Reduced PARP5B expression inhibited tumor growth, induced differentiation and apoptosis, and suppressed cell proliferation and metastasis. Moreover, the loss of PARP5B activated the ataxia telangiectasia and Rad3‐related (ATR) protein and diminished cancer stem cells. Notably, PARP5B‐deficient cells show compromised NHEJ‐mediated DSBs repair. These cells form a multiprotein complex at stalled replication forks, indicating the reliance on HR‐mediated repair at the replication foci. Additionally, treatment with low‐dose etoposide combined with XAV939 (a PARP5B inhibitor) induced senescence and apoptosis in human SCC cell lines, which was dependent on Nibrin, a DSBs repair protein [137].

PARP6 is a downstream target of small nucleolar RNA host gene 1 (SNHG1). In FaDu cells, SNHG1 suppression upregulates PARP6 expression. Both SNHG1 inhibition and PARP6 overexpression suppress the proliferation, migration, and invasion of FaDu cells. Additionally, they inhibit tumorigenicity in in vivo hypopharyngeal squamous cell carcinoma (HSCC) models. In HSCC, after either in vitro or in vivo SNHG1 inhibition and/or PARP6 overexpression, the protein levels of E‐cadherin significantly increase, whereas the levels of N‐cadherin, β‐catenin, and X‐ray repair cross‐complementing protein 6 (XRCC6) significantly decrease [138].

PARP6 is expressed at low levels in hepatocellular carcinoma (HCC), and its expression is negatively correlated with tumor differentiation. Additionally, in vitro and in vivo assays have shown that PARP6 silencing leads to increased proliferation, invasion, and migration of HCC cells. Conversely, increased PARP6 expression yields opposing effects. Gene chip analysis combined with experimental verification has confirmed that PARP6 inhibits XRCC6 expression by inducing its degradation, thereby suppressing the Wnt/β‐catenin pathway, which contributes to tumor suppression in HCC. Further mechanistic studies demonstrated that the ubiquitin ligase HDM2 interacts with PARP6 and XRCC6 and mediates PARP6‐dependent XRCC6 degradation [139].

The tumor‐suppressive role of PARP7 lies in limiting the cytoplasmic accumulation of interferogenic nucleic acids in malignant cells or their precursors [140]. In addition to suppressing type I IFN responses, PARP7 restricts the activation of oncosuppressive pathways coordinated by IRF1 and IRF3, which finally lead to caspase 8 activation and apoptotic cell death [140].

PARP10 expression promotes replication fork stability and inhibits replication stress, thereby promoting cell proliferation in vitro and tumor growth in vivo [141]. The loss of the monADP‐ribosyltransferase PARP10 significantly increases tumor migration and invasion by regulating epithelial–mesenchymal transition (EMT), and its tumor‐suppressive function in metastasis depends on its enzymatic activity. Mechanistically, PARP10 interacts with Aurora A and alters it through mono‐ADP‐ribosylation, thus inhibiting its kinase activity and regulating downstream signaling. In addition, PARP10 expression is low in metastatic HCC, compared with primary liver cancer and adjacent nontumor tissues [142].

Zhang et al. [143] reported that PARP11 is overactivated in response to adenosine and other factors that may exist in the immunosuppressive tumor microenvironment. Activated PARP11 stabilizes β‐transducin repeat‐containing protein (β‐TrCP), thereby accelerating the ubiquitination and degradation of interferon alpha and beta receptor subunit 1 (IFNAR1). Partial IFNAR1 loss impairs the cytolytic activity of CD8^+^ cytotoxic T lymphocytes and reduces their viability [144].

PARP12 acts as a tumor suppressor in HCC metastasis by regulating the stability of four and a half LIM domains protein 2 and the expression of transforming growth factor‐beta 1. Shao et al. [145] showed that PARP12 deficiency increased HCC migration and invasion by regulating the EMT, resulting in increased metastasis in vivo.

Joo‐Young et al. [146] reported that PARP16 plays a key role in cytochrome b5 reductase 3‐induced lung cancer apoptosis by increasing the ADP‐ribosylation of PERK and inositol‐requiring enzyme 1 alpha (IRE1α). In this study, ARTC1 (ART1) and ARTD15 (PARP16) were identified as ER‐resident ARTs that mediate the monoADP‐ribosylation of their substrates. ER stress activates both ARTC1 and PARP16, which regulate the unfolded protein response in the ER. Specifically, ARTC1 inactivates the molecular chaperone GRP78 through mono‐ADP‐ribosylation, thereby triggering ER stress by dissociating GRP78 from its interacting partners (e.g., PERK, IRE1α, and ATF6). Conversely, PARP16 mono‐ADP‐ribosylates PERK and IRE1α, thereby enhancing their enzymatic activity and amplifying downstream stress response [146].

### 4.2 Role in Neurological Disorders

The nervous system functions as a sophisticated “command center,” and its dysfunction can lead to a cascade of debilitating neurological disorders. The involvement of the PARP family in these disorders is highly complex. In neurodegenerative diseases, such as Alzheimer's disease and Parkinson's disease, PARP overactivation triggers a cascade of neuroinflammation and oxidative stress, finally accelerating neuronal damage and death.

Moderate PARP1 activation is central to maintaining homeostasis by facilitating DNA repair [39]. Conversely, aberrant and excessive PARP1 activation drives cell death in non‐neoplastic diseases. During the early stages of neurodegeneration, a controlled activation of PARP1 is initiated in response to low levels of DNA damage, which supports DNA repair. However, with disease progression, the accumulation of damaged DNA owing to pathological protein aggregates or other triggers induces PARP1 hyperactivation [40]. This, in turn, triggers PARP1‐mediated cell death, followed by subsequent neuroinflammatory responses, finally exacerbating neurodegenerative disease progression [132, 133, 134].

PARP2 exhibits dual inflammatory roles in the nervous system, depending on the context across brain injury models. In the organotypic hippocampal slice model exposed to oxygen‐glucose deprivation (OGD)—which predominantly exhibits apoptotic‐like features—PARP2 activation promotes the survival of CA1 pyramidal neurons. Conversely, in mixed cortical cultures subjected to OGD (where cell death is primarily necrotic), pharmacological inhibition of PARP2 reduces postischemic damage and enhances cell viability [171]. Furthermore, in experimental autoimmune encephalomyelitis, PARP2 knockout (PARP2^–^/^–^) mice demonstrate delayed disease onset and markedly reduced T helper 17 (Th17) lymphocytes, thus leading to attenuated central nervous system inflammation [172, 173].

PARP6, a neuron‐enriched PARP family member, is essential for neuronal function. The loss of its catalytic activity leads to detrimental effects. Vermehren‐Schmaedick et al. [147] developed a mouse model expressing a truncated variant of Parp6 (Parp6^TR^); this model lacks catalytic activity, compared with Parp6^WT^. Parp6^TR^ homozygous mice showed no apparent neurodevelopmental defects during embryogenesis; however, they succumbed postnatally. Therefore, PARP6 catalytic activity is critical for postnatal survival. They further identified PARP6 mutations in six patients with neurodevelopmental disorders, including cerebellar malformations, intellectual disability, and epilepsy. Among these, PARP6^C563R^—the most severe variant—resulted in the complete ablation of catalytic activity. Parp6^C563R^ expression in hippocampal neurons significantly impaired dendritic morphogenesis [147].

Wang et al. [148] demonstrated that PARP16 knockdown alleviated spatial memory deficits, reduced amyloid‐beta (Aβ) deposition, diminished neuronal apoptosis, and decreased proinflammatory cytokine production in *APPswe/PS1dE9* (APP/PS1) transgenic mice. In vitro experiments further suggested that PARP16 silencing mitigated Aβ‐induced neuronal damage and ER stress. Mechanistically, PARP16 functions as an RNA‐binding protein that stabilizes amyloid precursor protein (APP) mRNA, thereby preventing APP degradation and elevating protein levels, which exacerbate Alzheimer's disease pathology [65, 149].

### 4.3 Role in Diabetes and Metabolic Disorders

PARP1 activation has been implicated in both neuropathic and vascular alterations associated with diabetic retinopathy (DR) [174, 175]. Elevated glucose levels upregulate PARP1 expression, which mediates glucose‐induced vascular damage and inflammatory responses [41]. During DR pathogenesis, oxidative stress‐induced DNA strand breaks trigger PARP1 hyperactivation, thereby exacerbating these pathological processes [175]. Notably, high PARP1 mRNA and protein levels have been identified in the inner nuclear layer and ganglion cell layer of diabetic rat retinas [176]. Wang et al. [177] reported the dual regulatory role of PARP1 in hyperglycemia‐induced endothelial injury. In the early phase, PARP1 promotes endothelial repair by activating P53 and P53R2 pathways at 12 h postinjury; nonetheless, in the later phase, PARP1 activation triggers caspase‐3, particularly after 3 days of sustained hyperglycemia [178].

### 4.4 Role in Cardiovascular and Cerebrovascular Diseases

The cardiovascular and cerebrovascular systems serve as the “power pump” and “transport network,” respectively, and their integrity is vital for sustaining life. In ischemic injuries, such as myocardial infarction and cerebral infarction, PARP family members are robustly activated during ischemia‐reperfusion episodes. Though this activation initially supports cellular stress repair, its pathological overactivation paradoxically exacerbates tissue damage by triggering inflammatory cascades and endothelial dysfunction [43, 179]. In chronic conditions, such as hypertension and atherosclerosis, PARPs persistently modulate vascular smooth muscle cells (VSMCs) and endothelial cells, thereby driving disease progression. These findings underscore PARPs as molecular targets for the prevention and treatment of cardiovascular and cerebrovascular disorders.

PARP1 promotes the osteogenic transdifferentiation of VSMCs by upregulating key transcription factors, for example, runt‐related transcription factor 2 (RUNX2) and nuclear factor kappa‐light‐chain‐enhancer of activated B cells (NF‐κB). Pharmacological PARP1 inhibition protects against vascular mineralization and calcification [180]. Further studies revealed that PARP1 triggers polymerase gamma ubiquitination and degradation via PARylation modification, leading to mitochondrial dysfunction and activation of the Adora2a/Rap1 pathway, thereby inducing VSMC ferroptosis and vascular calcification, which can be reversed by PARP1 inhibition [181]. In inorganic phosphate (Pi)‐induced vascular calcification models, A10 VSMCs showed dysregulation of smooth muscle differentiation markers (e.g., smooth muscle 22‐alpha and α‐smooth muscle actin (α‐SMA)) and osteogenic markers (e.g., RUNX2 and alkaline phosphatase). Notably, MLN4924—a selective inhibitor of the NEDD8‐activating E1 enzyme—reversed these alterations and suppressed Pi‐induced calcium deposition, implicating PARP1's involvement in calcification [182].

PARP1 overexpression has been correlated with poor survival outcomes in acute myeloid leukemia (AML). Luedtke et al. [183] demonstrated that this association is particularly pronounced in fms‐like tyrosine kinase 3 (FLT3)‐mutated M4/M5 AML subtypes (a prognostic marker). Approximately 25% of AML cases showed PARP1 overexpression, driven by oncogenic pathways. PARPi (e.g., olaparib) exploit “synthetic lethality” in solid tumors, and this mechanistic rationale strongly supports their clinical evaluation in FLT3‐mutated AML subtypes [183].

In cardiac regeneration models, CPT1 inhibition reverses metabolic reprogramming and reduces PARP1‐mediated DUSP1 PARylation, leading to decreased p38 mitogen‐activated protein kinase (MAPK) phosphorylation and activation of adult cardiomyocyte proliferation post‐myocardial infarction [184].

Cai et al. [185] suggested that PARP2 mediates doxorubicin (DOX)‐induced cardiomyocyte senescence. PARP2 downregulation attenuated DOX‐triggered senescence, whereas its overexpression exacerbated this process [185]. Chen et al. [150] demonstrated that PARP9 overexpression reverses the antifibrotic effects of pirfenidone on angiotensin II‐induced fibrotic markers, including collagen I/III, α‐SMA, CTGF, and fibronectin. Gao et al. [151] identified that the CHAPIR‐PIWI‐like RNA‐mediated gene silencing 4 complex directly interacts with methyltransferase‐like 3, thereby blocking N6‐methyladenosine methylation of PARP10 transcripts and upregulating PARP10 expression. CHAPIR‐dependent PARP10 elevation promotes mono‐ADP‐ribosylation of glycogen synthase kinase‐3 beta, inhibiting its kinase activity. This cascade leads to the nuclear accumulation of nuclear factor of activated T cells and drives pathological cardiac hypertrophy [151].

PARP15‐dependent ADP‐ribosylation represents a genetically determined mechanism governing vascular responses to inflammation. Chan et al. [186] identified PARP15 as a novel genetic susceptibility factor in monoclonal gammopathy‐associated capillary leak syndrome. In human microvascular endothelial cells, PARP15 suppresses cytokine‐induced barrier disruption by ADP‐ribosylation of c‐Jun N‐terminal kinase (JNK)‐interacting protein 3 (the scaffold protein), thereby inhibiting p38 MAPK activation. Notably, mice expressing an enzymatically inactive PARP15^G628R^ mutant exhibited increased susceptibility to inflammation‐driven vascular leakage in a p38 MAPK‐dependent manner, compared with WT PARP15‐expressing controls [186]. PARP16 deficiency alleviates cardiac dysfunction, attenuates transverse aortic constriction‐induced cardiac hypertrophy/fibrosis, and reduces phenylephrine‐induced cardiomyocyte hypertrophic responses. Su et al. [152] demonstrated that PARP16 binds to IRE1α and facilitates its ADP‐ribosylation, which mechanistically activates the IRE1α–X‐box binding protein 1–GATA4 axis to drive pathological cardiac hypertrophy.

### 4.5 Role in Inflammatory Diseases

Inflammation has a dual role in human pathophysiology: though it serves as a protective alert system against pathogens, dysregulated inflammation paradoxically promotes chronic disease progression. The PARP family functions as a molecular rheostat in inflammatory disorders. PARPs amplify inflammatory cascades through post‐translational modification of key signaling molecules (e.g., NF‐κB and activator protein 1), thus sustaining a proinflammatory microenvironment. Specifically in the intestinal mucosa. In contrast, PARPs regulate immune cell recruitment (e.g., neutrophil chemotaxis) and effector functions (e.g., macrophage polarization), thereby modulating the resolution or persistence of inflammation. These dual roles position PARP‐targeted therapies as a precision strategy to eliminate pathological inflammation while preserving physiological immune surveillance.

Stanniocalcin‐1 (STC1)—a novel mediator of PARP1‐dependent necroptosis—is upregulated under inflammatory conditions and modulates oxidative stress‐induced programmed cell death. Zhu et al. [35] demonstrated that STC1‐overexpressing cells exhibited enhanced necroptosis and elevated proinflammatory cytokine production, whereas STC1‐knockout cells showed reduced cell death. Mechanistically, STC1 directly interacts with PARP1, which activates the JNK pathway via the formation of the PARP1–JNK complex. Pharmacological inhibition of PARP1 or JNK significantly attenuated STC1‐driven cell death and inflammation. Importantly, adeno‐associated virus‐mediated restoration of Stc1 and Parp1 expression exacerbated dextran sodium sulfate‐induced colitis in *Stc1^INT‐KO^* mice, confirming the pathological synergy between STC1 and PARP1 in vivo [35]. Pyrazinamide (PZA) has been identified as an anti‐inflammatory molecule that attenuates cytokine signaling and lesion activity in tuberculosis (TB). PARP1 functions as a major proinflammatory regulator activated during TB infection and as a host target mediating PZA's immunomodulatory functions. PZA suppresses PARP1 enzymatic activity in both macrophages and murine models, reversing TB‐induced PARP1 hyperactivation in infected lungs to baseline levels. Crucially, using PZA‐resistant mutants, researchers have demonstrated that PZA's immune‐modulatory effects are PARP1‐dependent but bactericidal activity‐independent. Furthermore, adjunctive PARP1 inhibitors reduced pulmonary inflammation and lesion size in mice, suggesting a therapeutic strategy to mitigate lung injury and shorten TB treatment duration [36].

Researchers have identified elevated PARP2 mRNA expression in human psoriatic skin lesions. Functional investigations using both murine and human psoriatic cellular models suggested that PARP2 deficiency ameliorates disease pathology. Specifically, PARP2 knockout attenuated imiquimod‐induced psoriasiform dermatitis in mice. In human keratinocytes, PARP2 silencing suppressed hyperproliferation, preserved terminal differentiation markers, and reduced inflammatory mediator production after stimulation with psoriasis‐associated cytokines (IL‐17A and TNFα). Mechanistically, PARP2 deficiency upregulated aromatase in both *Parp2‐KO* mice epidermis and PARP2‐deficient human keratinocytes, leading to elevated estradiol levels. This activity inhibited NF‐κB signaling and subsequent keratinocyte‐driven inflammation [173]. In T cell‐specific PARP2 knockout mice, reduced TNFα production and diminished IL‐17 levels confirmed the protective role of PARP2 inhibition against intestinal inflammation. Additionally, PARP2 downregulation in T cells decreased oxidative‐nitrosative stress and PARP1 hyperactivation, concomitant with altered extracellular signal‐regulated kinase and NF‐κB signaling [154].

Hou et al. [187] suggested that PARP5A and its binding partner RNF146 regulate necroptosis. After the initiation of TAX1BP1‐induced necroptotic signaling, PARP5A and RNF146 undergo liquid–liquid phase separation through multivalent interactions, forming dynamic condensates. These condensates facilitate PARylation and PARylation‐dependent ubiquitination (PARdU), which activates RIPK1 at the K376 residue in mouse embryonic fibroblasts. This post‐translational modification promotes the proteasomal degradation of kinase‐active RIPK1, thereby suppressing necroptosis. Hence, PARP5A–RNF146 condensate axis regulates K376 PARdU modification through phase separation‐driven mechanisms, establishing a novel cell death checkpoint for necroptosis control [187].

PARP9 exerts proinflammatory effects by augmenting IFNγ responses, whereas PARP14 exhibits anti‐inflammatory properties by suppressing IFNγ signaling and enhancing IL‐4‐mediated pathways. Therapeutically, PARP9 inhibition and/or PARP14 activation attenuate macrophage‐mediated vascular damage [62]. Zhu et al. [156] identified PARP9 as a gene exhibiting concordant DNA methylation and mRNA expression changes between patients with rheumatoid arthritis and healthy controls. This regulatory role of PARP9 was validated experimentally: hypermethylation at specific loci correlated with decreased PARP9 expression. Furthermore, functional analyses demonstrated that PARP9 knockdown in Jurkat cells suppressed proliferation and lowered the expression of primary activation markers [156].

Deng et al. [157] demonstrated that PARP12 suppresses mitophagy and exacerbates OA progression in both human OA cartilage and sodium iodoacetate‐induced rat OA models. Mechanistically, PARP12 interacts with interferon‐stimulated gene 15 to enhance interferon‐stimulated gene (ISG)ylation of mitofusin 1/2 (MFN1/2), which reduces their ubiquitination and SUMOylation. This post‐translational modulation inhibits PINK1/Parkin‐dependent mitophagy in chondrocytes, finally promoting cartilage degradation. Furthermore, inflammatory cytokine‐induced activation of IRF1 is essential for PARP12 upregulation. This is because IRF1 directly binds to the PARP12 promoter to drive its transcription. Pharmacological inhibition of PARP12 using XAV‐939 attenuated OA pathogenesis both in vitro and in vivo. Clinically, PARP12 expression levels correlated with OA severity, positioning it as a predictive biomarker and novel therapeutic target for modulating mitophagy in OA [157].

### 4.6 Role in Infections and Viral Diseases

Regarding microbial invasions, such as bacterial and viral infections, the immune system triggers a rapid defense mechanism, and the PARP family participates in the immune response. In the early stages of viral infection, PARPs recognize viral nucleic acids, initiating an innate immune response and establishing a frontline defense against viral invasion. With infection progression, PARPs play a dual role in regulating host cell survival and the viral replication cycle: they either promote the elimination of the virus or are hijacked by viruses. Analyzing its dual‐faced role in infection and viral diseases is central to guiding anti‐infective strategies.

Viral infections suppress host immune competence and compromise genomic stability [188, 189]. DNA viruses induce reactive nitrogen species‐dependent DNA damage, activating DNA‐dependent protein kinase (DNA‐PK). Activated DNA‐PK phosphorylates PARP1 at Thr594, promoting its cytoplasmic translocation and suppression of antiviral immunity both in vitro and in vivo. Mechanistically, cytoplasmic PARP1 binds to cyclic GMP‐AMP synthase (cGAS) at Asp191 and catalyzes its ADP‐ribosylation, thereby inhibiting cGAS's capacity to bind DNA [158]. ADP‐ribosylation events critically regulate the life cycle of severe acute respiratory syndrome coronavirus 2 (SARS‐CoV‐2) and host inflammatory responses. In patients with coronavirus disease 2019, elevated oxidative stress and robust PARylation have been detected across pulmonary cell types, which correlate inversely with lymphopenia. The PARPi rucaparib reduces SARS‐CoV‐2 infection by binding to a conserved 493 to 498 amino acid region within the spike‐angiotensin‐converting enzyme 2 interface, thereby blocking viral attachment. At pharmacologically relevant concentrations, rucaparib‐mediated PARP1 inhibition downregulates spike protein‐ and viral RNA‐induced cytokine hyperexpression [190, 191].

**FIGURE 5:**
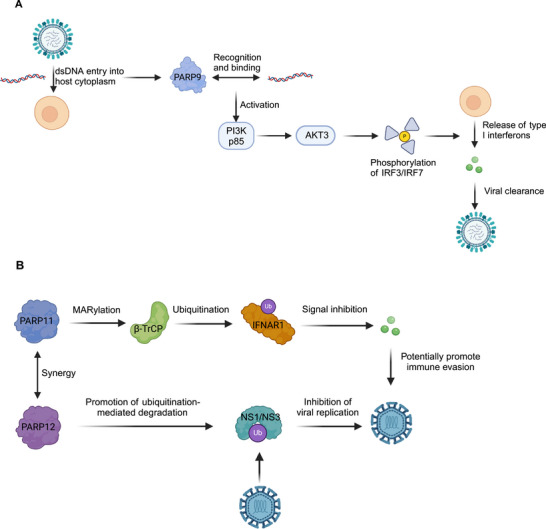
Role of PARP9 and PARP11 in antiviral responses. (A) PARP9 directly binds to dsRNA from RNA viruses, activates the PI3K/AKT3 signaling pathway, induces phosphorylation of interferon regulatory factors IRF3/IRF7, and drives the production of type I interferons (IFN‐α/β). These steps eliminate viral infections. (B) PARP11 and PARP12 synergistically promote the ubiquitination‐mediated degradation of ZIKV NS1/NS3 proteins, directly inhibiting viral replication. Additionally, they mono‐ADP‐ribosylate the ubiquitin E3 ligase β‐TrCP, inducing the degradation of the interferon receptor IFNAR1, thereby suppressing antiviral signaling. This figure shows the dual function of PARP11 in viral clearance and immune regulation. *Abbreviations*: PARP, poly (ADP‐ribose) polymerase; PI3K, phosphoinositide 3‐kinase; dsRNA, double‐stranded RNA; IRF, interferon regulatory factor; ZIKV, Zika virus; IFN, interferon; β‐TrCP, β‐transducin repeat‐containing protein; and IFNAR1, interferon alpha/beta receptor 1. Image created with BioRender.com, with permission.

Zhang et al. [84] first reported that PARP9 recognizes and binds double‐stranded RNA from RNA viruses. This interaction activates the PI3K p85 subunit and AKT3 signaling pathway, leading to the phosphorylation of IRF3 and IRF7. These events culminate in IFN‐I production, thereby enhancing resistance to RNA viral infections and facilitating viral clearance (Figure 5A). Notably, this study confirmed PARP9 as a mitochondrial antiviral‐signaling protein‐independent, noncanonical RNA sensor, advancing the current understanding of alternative RNA‐sensing mechanisms in innate immunity [84].

Krieg et al. [160] demonstrated that PARP10‐dependent MARylation inhibits the enzymatic activity of the nonstructural protein 2 protease, which is essential for Chikungunya virus replication. This MARylation‐mediated suppression disrupts polyprotein processing, thereby blocking viral replication. Importantly, the macrodomain of nonstructural protein 3 (Nsp3) reverses this inhibitory effect by hydrolyzing MARylation. Consequently, the loss of MAR hydrolase activity in nsP3 disrupts polyprotein maturation, further halting viral propagation [160].

Researchers have identified PARP11 as an ISG that functions synergistically with PARP12 to restrict Zika virus (ZIKV) replication by degrading NS1 and NS3 proteins. Upon IFNα/IFNβ stimulation or ZIKV infection, PARP11 expression is upregulated in WT cells but not in IFNAR1 knockout (IFNAR1^−^/^−^) cells, indicating its dependence on interferon receptor signaling. Functionally, ZIKV replication is suppressed in PARP11‐expressing cells but enhanced in PARP11^−/−^ cells. Mechanistically, PARP11 inhibits the enzymatic activity of ZIKV [9], while its interaction with PARP12 synergistically enhances PARP12‐mediated proteasomal degradation of NS1 and NS3 proteins [161] (Figure 5B). Furthermore, PARP12 has shown antiviral responses against coronaviruses; however, the activity of additional PARP family members is required to prevent Mac1‐mutant viruses from causing severe disease in vivo [192].

PARP11 mono‐ADP‐ribosylates the ubiquitin E3 ligase β‐TrCP, facilitating IFNAR1 recruitment and its ubiquitination‐dependent degradation. This PARP11‐driven pathway attenuates IFN signaling and impairs host defense mechanisms. These results elucidate a novel PARP11‐mediated signaling axis and identify ADP‐ribosylation as a critical mechanism regulating IFN activity and antiviral efficacy [162]. PARP13 establishes a cellular environment favorable to antiviral defense by limiting its translation, regulating mRNA entry, and enhancing ISG expression. This mechanism involves the acylation of ISGs [164]. PARP14, the major ADP‐ribosyltransferase upregulated by IFNγ, has its protein stability regulated by the PARP9/DTX3L complex. Moreover, this modification can be hydrolyzed by the macrodomain of SARS‐CoV‐2 Nsp3. These findings enable understanding the role of ADP‐ribosylation in antiviral signaling and elucidate viral macrodomains as novel targets for antiviral therapy [159].

## 5 Conclusions and Perspectives

### 5.1 Future Challenges and Directions in PARP‐Targeted Therapies

PARP‐targeted therapies have demonstrated substantial clinical potential. The mechanism of action of PARPi in cancer treatment has been validated through preclinical animal models and clinical trials [193]. Notably, PARPi, such as olaparib, have demonstrated significant efficacy in cancer treatment with BRCA mutations, including ovarian cancer [194] and breast cancer [195] (Table 2).

**TABLE 2:** PARP inhibitors: mechanisms, preclinical models, and clinical trial progress.

PARP inhibitor	Targets	Mechanism	Indications	Preclinical animal experimental model	Clinical research phase	References
Olaparib	PARP1/2	Synthetic lethality, PARP trapping	Breast cancer; recurrent epithelial ovarian cancer; fallopian tube cancer; primary peritoneal cancer; and ovarian cancer	BRCA‐mutant mice and canine prostate cancer models	III	[196, 197, 198]
Niraparib	PARP1/2	Synthetic lethality	Recurrent epithelial ovarian cancer; fallopian tube cancer; and primary peritoneal cancer	Ovarian cancer xenograft model	III	[194, 199]
Rucaparib	PARP1/2/3	Synthetic lethality	Pancreatic cancer, BRCA‐mutated advanced ovarian cancer	Transgenic mouse model of pancreatic cancer	II	[200, 201]
Talazoparib	PARP1/2	High PARP trapping capacity	BRCA‐mutated breast cancer; HRR‐deficient prostate cancer	Breast cancer organoid model	III	[202, 203]
Fuzuloparib	PARP1	Synthetic lethality	Treatment of high‐grade, platinum‐sensitive, recurrent ovarian cancer; prostate cancer	Prostate cancer PDX model	II	[204, 205, 206]
Veliparib	PARP1/2	PARP trapping	Breast cancer; lung cancer; and glioblastoma	Triple‐negative breast cancer xenograft model	III	[207, 208, 209, 210]

Beyond BRCA‐mutant tumors, emerging clinical evidence supports PARPi efficacy in selected non‐BRCA populations. For example, the NORA trial showed that niraparib significantly improved median overall survival in gBRCA WT patients with platinum‐sensitive recurrent ovarian cancer [211, 212]. Moreover, the TRITON3 trial confirmed talazoparib's benefit in non‐BRCA HRR‐mutated metastatic CRPC, such as *PALB2*‐mutated cases, extending the median radiographic progression‐free survival to 11.2 months, compared with 6.4 months with standard therapy (hazard ratio = 0.50) [202, 213, 214, 215].

The mechanisms of action of PARPi in monotherapy primarily involve synthetic lethality and PARP trapping. Synthetic lethality refers to PARP inhibition that blocks the DNA damage repair pathway in tumor cells, effectively prolonging patient survival [52, 216]. PARP trapping occurs when PARPi binds to the NAD^+^ binding pocket of PARP1 (and/or PARP2), inducing conformational changes that stabilize the reversible dissociation of the DNA–PARP complex. This PARP trapping on DNA is known as DNA–PARP complex “trapping” [217]. Commonly used PARPi are catalytic inhibitors, and their ability to capture the DNA–PARP complexes varies. The trapping potency is ranked in descending order as follows: talazoparib > niraparib > olaparib = rucaparib > veliparib [218].

The combined use of PARPi has expanded its therapeutic potential, with primary mechanisms including coadministration with antiandrogens, radiotherapy/chemotherapy, or immune checkpoint inhibitors [205]. Synthetic lethality may be attributed to not only the inability of tumor cells to repair unresolved transcription‐replication conflicts but also PARP trapping. PARP1 functions synergistically with TIMELESS and TIPIN to protect the early S phase from these conflicts. Inhibiting transcriptional elongation confers resistance to PARPi in HR‐deficient cells, whereas TIPIN depletion is lethal to these cells. Hence, PARP enzymatic activity may suffice under certain therapeutic settings [219].

Despite their promising results, PARP‐targeted therapies face major limitations. One of the common limitations is drug resistance. Tumor cells may activate alternative DNA repair pathways (e.g., alternative NHEJ) to bypass PARP inhibition‐induced repair defects, leading to therapeutic resistance [220, 221, 222].

To this end, several strategies to overcome resistance are being explored:

- The development of next‐generation PARPi, such as thioparib, which exhibits stronger PARP1 enzymatic inhibition (IC_50_ = 0.4 nM) and DNA trapping (EC_50_ = 25 nM) than Olaparib. Additionally, it suppresses HR repair and activates type I interferon signaling [223].
- Combination therapies, such as coadministration with ATR inhibitors, including camonsertib, to block the activated ATR–CHK1 axis in resistant cells [224].
- The use of epigenetic modulators, such as 5‐azacytidine, to disrupt DNA repair by reducing DNA methyltransferase 1‐dependent repair factor recruitment [225].
- The utilization of PROTACs, such as SK‐575, to degrade PARP1 and bypass mutation‐driven resistance [226].
- Rechallenge strategies of readministering PARPi after a drug‐free interval, which shows partial responses in patients with transient resistance because of the clonal evolution of resistant subpopulations [227].

Additionally, adverse effects of PARPi, including myelosuppression (anemia, thrombocytopenia) and gastrointestinal toxicity, restrict the dosage intensity and treatment duration, compromising quality of life and treatment compliance [228]. Current patient stratification based on HRR deficiency (e.g., BRCA mutations) remains inadequate, because subsets of HRR‐proficient tumors still benefit from PARPi. This finding necessitates more precise predictive biomarkers, such as replication stress scores, to optimize patient selection and treatment outcomes.

### 5.2 Future Research Priorities

#### 5.2.1 Context‐Specific Mechanisms Underlying PARPs in Pathological States

Future studies must deepen the understanding PARP's noncanonical roles in nonmalignant diseases. For instance, PARP14 regulates Th17 cell differentiation and promotes autoimmune diseases [229]. The PARP9/DTX3L complex suppresses antiviral immune responses by modifying host proteins during infection [230, 231]. Though PARPs have been extensively studied in diabetic neuropathy, their mechanisms in other metabolic disorders (e.g., diabetic nephropathy) remain poorly characterized. Future studies utilizing tissue‐ and disease‐specific PARP interactomes may uncover novel pathological pathways.

A comprehensive understanding of PARP dysregulation across diverse neurodegenerative diseases is warranted. Although PARP hyperactivation drives neuronal injury, the underlying signaling pathways and regulatory networks remain poorly defined. Systematic investigation into how PARPs contribute to Alzheimer's disease and Parkinson's disease, particularly via mitochondrial function, neuroinflammatory responses, and neurotransmitter metabolism, will provide the groundwork for novel therapeutic targets.

#### 5.2.2 Development of Novel PARP Modulators and Preclinical Perspectives

The development and preclinical assessment of novel PARP modulators hold great promise. Currently, most PARPi are small‐molecule inhibitors. In the future, research should explore novel modalities, such as nucleic acid aptamers and nanobodies [228], which may offer higher specificity, reduced systemic toxicity, and enhanced drug delivery. Preclinical studies should evaluate their pharmacodynamics, pharmacokinetics, and safety in disease models, laying the groundwork for subsequent clinical translation. Future development should extend beyond the traditional catalytic inhibition model and explore the following directions:

1. *Allosteric inhibitors* [232]: Targeting noncatalytic PARP domains (e.g., the histone PARylation factor 1 interaction sites) to minimize off‐target effects [233, 234].
2. *Bifunctional molecules* [226, 235]: Designing PARP1–targeting proteolysis‐targeting chimeras (PARP1–PROTACs) to induce ubiquitination‐dependent PARP1 degradation, overcoming resistance to catalytic inhibitors [37, 38].
3. *Tissue‐selective delivery systems* [236, 237, 238]: Utilizing nanocarriers or antibody–drug conjugates (ADCs) (e.g., tumor antigen‐directed ADCs) to enhance tumor specificity and reduce systemic toxicity.

## Author Contributions

Pengyuan Lei and Wenfeng Li drafted the initial manuscript. Jinhua Luo, Nanxin Xu, and Yahe Wang performed literature curation and data collection. Dafei Xie and Hua Guan provided supervision and project administration. Xin Huang revised the manuscript. Bo Huang and Pingkun Zhou conducted critical review and validation. All authors read and approved the final manuscript, with original figures created by Wenfeng Li.

## Conflicts of Interest

All authors declare no conflicts of interest.

## Ethics Statement

The authors have nothing to report.

## Data Availability

The authors have nothing to report.
